# Atheroprotective Effect of Oleoylethanolamide (OEA) Targeting Oxidized LDL

**DOI:** 10.1371/journal.pone.0085337

**Published:** 2014-01-20

**Authors:** Angran Fan, Xiaofeng Wu, Huijuan Wu, Long Li, Rui Huang, Yueyong Zhu, Yan Qiu, Jin Fu, Jie Ren, Chenggang Zhu

**Affiliations:** 1 Department of Medical Science, Medical College, Xiamen University, Xiamen, Fujian, China; 2 Division of Liver Disease, the First Affiliated Hospital, Fujian Medical University, Fuzhou, Fujian, China; 3 School of Life Sciences, Fudan University, Shanghai, China; Sapienza University of Rome, Italy

## Abstract

Dietary fat-derived lipid oleoylethanolamide (OEA) has shown to modulate lipid metabolism through a peroxisome proliferator-activated receptor-alpha (PPAR-α)-mediated mechanism. In our study, we further demonstrated that OEA, as an atheroprotective agent, modulated the atherosclerotic plaques development. *In vitro* studies showed that OEA antagonized oxidized LDL (ox-LDL)-induced vascular endothelial cell proliferation and vascular smooth muscle cell migration, and suppressed lipopolysaccharide (LPS)-induced LDL modification and inflammation. *In vivo* studies, atherosclerosis animals were established using balloon-aortic denudation (BAD) rats and ApoE^-/-^ mice fed with high-caloric diet (HCD) for 17 or 14 weeks respectively, and atherosclerotic plaques were evaluated by oil red staining. The administration of OEA (5 mg/kg/day, intraperitoneal injection, i.p.) prevented or attenuated the formation of atherosclerotic plaques in HCD-BAD rats or HCD-ApoE^−/−^ mice. Gene expression analysis of vessel tissues from these animals showed that OEA induced the mRNA expressions of PPAR-α and downregulated the expression of M-CFS, an atherosclerotic marker, and genes involved in oxidation and inflammation, including iNOS, COX-2, TNF-α and IL-6. Collectively, our results suggested that OEA exerted a pharmacological effect on modulating atherosclerotic plaque formation through the inhibition of LDL modification in vascular system and therefore be a potential candidate for anti-atherosclerosis drug.

## Introduction

Atherosclerosis is a chronic inflammatory disease whose occurrence may be attributed to endothelial cell activation, low density lipoprotein (LDL) modification, macrophage chemotaxis, and vessel smooth muscle cell migration [1]. This inflammatory process leads to the development of complex lesions that protrude toward the arterial lumen and form plaques. Among many genetic and environmental causes, the accumulation of modified LDL [2], such as oxidized LDL (ox-LDL), and the recruitment of monocyte-derived macrophages at the arterial subendothelial spaces [3] are the key factors leading to the development of atherosclerotic lesion [1], [4].

Peroxisome proliferator-activated receptor-alpha (PPAR-α), a ligand-activated transcription factor, has emerged as a popular drug target for hyperlipidemia and inflammation in recent years [5]–[7]. Activation of PPAR-α by its agonists can reduce plasma lipid contents [8]–[10], improve glucose tolerance [7], [11]–[14] and attenuate inflammatory process [15]–[17]. Thus, PPAR-α agonists may be effective anti-hyperlipidemia or anti-inflammatory agents in terms of their effects on the lipoprotein profile and their anti-inflammation properties. Additionally, the broad expression of PPAR-α in vascular cells, including vascular endothelial cells, macrophages [18], and vascular smooth muscle cells [19], implicates that it might play a role in atherosclerotic lesion development. Fibrate, a classic PPAR-α agonist, modulates the expression of several genes that results in the decrease of monocyte recruitment [20], the inhibition of vascular smooth muscle cell migration and proliferation [21], and the acceleration of lipid removal from macrophages [22]. These atheroprotective effects, together with the lipid-lowering properties, are the mechanisms underlining the anti-atherosclerosis functions of the PPAR-α agonists.

Previous works has revealed that oleoylethanolamide (OEA), a gut-endocrine fat-derived lipid, is an endogenous ligand of PPAR-α [23]–[25]. Administration of OEA as a pharmacologic drug can cause (i) stimulation of lipolysis [26], [27]; (ii) induction of β-oxidation [23], [28]; and (iii) increase of fatty acid uptake [23], [24], [29] through the activation of PPAR-α signaling pathway. In present studies, we investigated the effect of OEA on the course of atherosclerosis development in clinically relevant animal models, i.e., high-caloric diet (HCD)-induced atherosclerosis in arterial denudated rats and HCD-induced atherosclerosis in ApoE null-mice (ApoE^−/−^). Our data elucidated a relation between PPAR-α signaling and atherogenesis, through which OEA may exert its therapeutic effect on atherosclerosis.

## Methods

### Animals

Sprague-Dawley (250–300 g) rats were purchased from Shanghai Laboratory Animal Center (Shanghai, China); Balb/c mice (4 week-old), C57/BL6 wild-type mice and ApoE^−/−^ mice (4 week-old) were purchased from Beijing University Laboratory Animal Center (Beijing, China). Animals were maintained on a 12-h light/dark cycle (on at 6∶00 and off at 18∶00) with free access to water and food (Research diet, Shanghai, China) *ad libitum* except indication. ApoE^−/−^ mice were randomly divided into 3 groups (n = 6–8/group) as follows: (i) group 1 received normal diet (ND); (ii) group 2 received high-caloric diet (HCD) (2% cholesterol, Research diet) following the vehicle treatment (saline/polyethylene glycol/Tween-80, 90/5/5, 1 ml/kg/day, 14 days, i.p.); and (iii) group 3 received HCD following OEA treatment (5 mg/kg/day, 14 days, i.p.). Drug was administered at the same time when animal received HCD. Animals were anesthetized with i.p. injection of ketamine (100 mg/kg)/xylazine (10 mg/kg) and sacrificed by decapitation. All procedures met the Guide for the care and use of laboratory animals published by the US National Institutes of Health (NIH Publication, 8th Edition, 2011) and were approved by Xiamen University Animal Care and Use Committees in China.

### Surgery

Rats were anesthetized with i.p. injection of ketamine (100 mg/kg) and xylazine (10 mg/kg). The left carotid artery was exposed up to its bifurcation. A Fogarty F2 Arterial Embolectomy Catheter (Edwards Lifesciences, Irvine, CA, USA) was introduced into the carotid artery and passed along to the abdominal aorta. Once the catheter was in place, the balloon was inflated by injection of 0.1 ml saline and pulled back/forward in the aorta 3 times, thereby causing vascular endothelial denudation. The balloon was deflated and the catheter was removed thereafter. Sham-operated animals went through the same procedure without balloon inflation. Animals were randomly divided into 3 groups as follows: (i) received normal diet, (ii) received HCD following vehicle treatment (1 ml/kg/day, 17 days, i.p.), and (iii) received HCD following OEA treatment (5 mg/kg/day, 17 days, i.p.). Sham-operated animals were given HCD with vehicle treatment as controls.

### Chemicals

OEA was synthesized in our lab as previously described [30]. MK866 was purchased from Cayman Chemical (Ann Arbor, MI, USA) while other chemicals were sourced from Sigma-Aldrich (Shanghai, China).

### Cell culture

RAW264.7 cells, human umbilical vein endothelial cells (HUVEC), and rat vascular smooth muscle cells (VSMC) were purchased from American Tissue Culture Collection (Beijing Zhongyuan Limited, Beijing, China) and cultured in Dulbecco's modified essential medium (DMEM) (Invitrogen, Shanghai, China) supplemented with 10% Fetal Bovine Serum (FBS) (Invitrogen).

Primary macrophage isolation: 4-week-old Balb/c mice were intraperitoneally (i.p.) injected with 2 ml of 3% sterile thioglycollate medium (Sigma-Aldrich). On the 4^th^ day after administration, primary macrophages were harvested by peritoneal lavage with 1× PBS (pH 7.5). Cells were collected by centrifugation and cultured in DMEM supplemented with 10% FBS and 2 mM glutamine (Sigma-Aldrich).

### *In vitro* cell assays

#### Annexin V flow cytometry assay

5×10^5^ HUVEC cells were placed on 6-well plate and cultured for 24 hrs with either vehicle or drugs. Cells were stained with Annexin V and PI staining kit (BD Pharmingen, Shanghai, China) following the manufacturer's instruction and cell apoptosis was analyzed by BD FACScan system (BD Pharmingen).

#### Cell proliferation assay

Cell viability was analyzed by a Cell Counting Kit-8 (CCK-8) (Dojindo Molecular Technologies, Shanghai, China). The HUVECs were plated on 96-well plates at a density of 5,000 cells per well and cultured for 12 hrs. Cell media were removed; the cells were incubated with tetrazolium reagent for 1 hr and then washed with 1×PBS. Colorimetric dye was detected for absorbance at wavelength 450 nm in spectrophotometer. The data were obtained from replicated experiments.

#### Transmigration experiments

Cell migration assays were performed with Transwell® Membrane (12 mm diameter, 8 μm pore size, Millipore, Shanghai, China). 1×10^5^ VSMC cells were plated on Transwell® Membranes inserted in 12-well plates and cultured overnight. Cells were treated with drugs for another 24 hrs. Non-migrating cells were wiped out by wet cotton swabs from upper membrane. Transwell® Membrane Inserts were transferred to hematoxylin stain solution for 3 mins and then rinsed in running tap water. The cells migrated to the reverse of membrane were counted in cytometer.

### RNA isolation and cDNA synthesis

Total RNA was extracted by TRIzol™ (Invitrogen) following the manufacturer's instruction and quantified with spectrophotometer (Beckman Coulter, Shanghai, China). cDNA was synthesized from 1 μg of total RNA with ReverTra Ace qPCR RT Kit (TOYOBO, Shanghai, China) following the manufacturer's instruction.

### Real-time quantitative PCR

Real-time quantitative PCR was performed by SYBR Premix Ex Taq™ GC (TaKaRa, Dalian, China) with ABI PRISM 7700 sequence detection system (Applied Biosystems, Foster City, CA). We designed the primer sets using the Primer Express software based on gene sequences available in GenBank database. The primers for mouse/rat genes were synthesized in accordance with the sequences shown in Table S1 (Sangon Biotech, Shanghai, China). The RNA expression levels were normalized by using glyceraldehyde 3-phosphate dehydrogenase (GAPDH) as an internal standard.

### Biochemical analyses

Blood was collected via left cardioventricle and serum was separated by centrifugation (1500×g, 30 min). Total cholesterol and triglyceride were determined using Total Cholesterol Assay Kit and Triglyceride Assay Kit (Kangtai Clinical Reagent, Beijing, China), respectively. HDL was determined using an High Density Lipoprotein Cholesterol Assay Kit (Huili, Changchun, China). LDL and ox-LDL were determined using Elisa kit (Uscn, Wuhan, China). TNF-α was detected by ELISA Kit (NeoBioscience Technology Company, Beijing, China).

### Histology

For atherosclerotic lesion staining, entire aortas were dissected, opened longitudinally, submerged into Oil-red O (Sigma-Aldrich) for 10 mins, differentiated in 85% propylene glycol solution for 3 mins, and rinsed with 1×PBS for 3 times. Images were taken with digital morphometric (Nikon, Shanghai, China).

For histology staining, aortas were removed, fixed quickly in Bouin's fixative solution (Sigma-Aldrich), and embedded in paraffin wax. 5 μm thick sections were cut by Sliding microtome Leica SM2010 R (Leica, Shanghai, China). Sections were dehydrated by gradient ethanol and stained with hematoxylin and eosin (H&E). Images were captured by microscope (Nikon).

### Statistical analyses

Results were expressed as the mean ± S.E.M of n separate experiments. The significance of the differences between groups was evaluated by one-way ANOVA followed by Dunnett's test for multiple comparisons.

## Results

### OEA reduced cell apoptosis and migration

**Figure 1:**
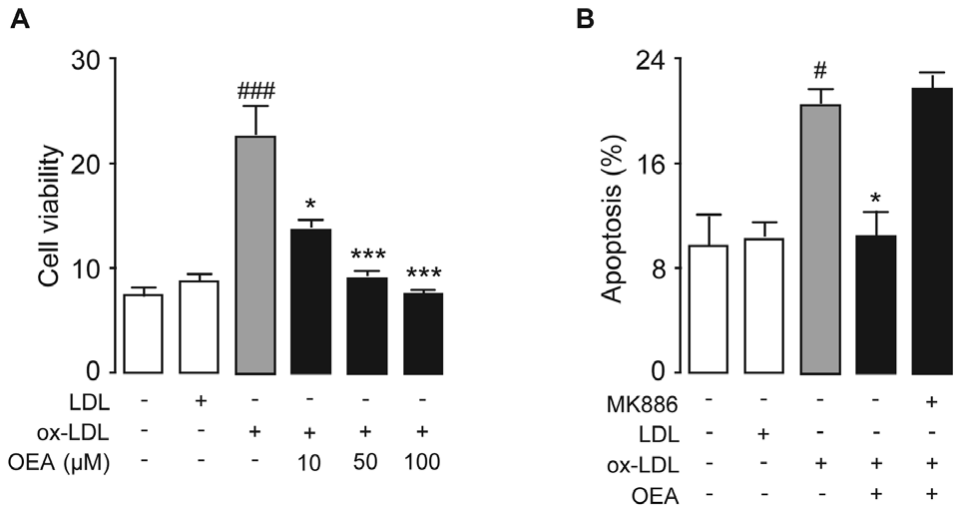
OEA reversed vascular endothelial cell proliferation and apoptosis induced by ox-LDL. (A) OEA dose-dependently reduced ox-LDL-induced cell proliferation as evaluated by CCK-8 assay. (B) The effect of OEA with or without MK886 on cell apoptosis, assessed by flow cytometer, in HUVEC cells treated with LDL or ox-LDL; Vehicle, 0.1% DMSO; LDL, 50 μg/ml; ox-LDL, 50 μg/ml; OEA 50 μM or concentration in μM; MK886, 10 μM. ^#^ p<0.05, ^###^ p<0.001 vs vehicle group; * p<0.05, *** p<0.001 vs ox-LDL group; one-way ANOVA, n = 6–8.

During the atherosclerotic development, vascular cells are the primary targets being attacked, causing vascular endothelial cell dysfunction, vascular cell proliferation and smooth muscle cell migration, which further lead to pathological lesions. We first investigated the effect of ox-LDL on the proliferation and apoptosis of HUVEC cells (Fig. S1). HUVECs were treated with ox-LDL (50 μg/ml) up to 48 hrs, and cell proliferation and apoptosis were analyzed at multiple time points. HUVEC proliferation was induced by ox-LDL as early as 12 hrs of the treatment, which was maintained in peak through 24 hrs, then was started to decrease after 36 hr of treatment. In contrast, the ox-LDL-induced apoptosis started only after 48 hrs of the ox-LDL stimulation, suggesting the over-proliferation induced by ox-LDL in HUVECs was the trigger of the cell apoptosis. Then we investigated the effects of OEA on vascular cell proliferation, apoptosis as well as migration. HUVEC cells were treated by LDL (50 μg/ml) or ox-LDL (50 μg/ml) with or without OEA (10, 50, 100 μM) for 24 hrs and cell proliferation assay showed an increase of cell viability by ox-LDL treatment, which was dose-dependently inhibited by OEA (Fig. 1A). Ox-LDL-induced over-proliferation of HUVEC cells could further lead to increased apoptosis, and cell death was detected by double-immunostaining of Annexin-V and PI. Flow cytometer analysis revealed that cell apoptosis was observed when HUVEC cells were treated with ox-LDL (Fig. 1B). However, ox-LDL-induced apoptosis in HUVEC cells could be blocked by 50 μM OEA (Fig. 1B), which was an effect mediated through PPAR-α, and PPAR-α antagonist MK886 (10 μM) reversed the effect of OEA. In contrast, LDL had neither effect on apoptosis nor viability on HUVEC cells (Fig. 1A–B).

**Figure 2:**
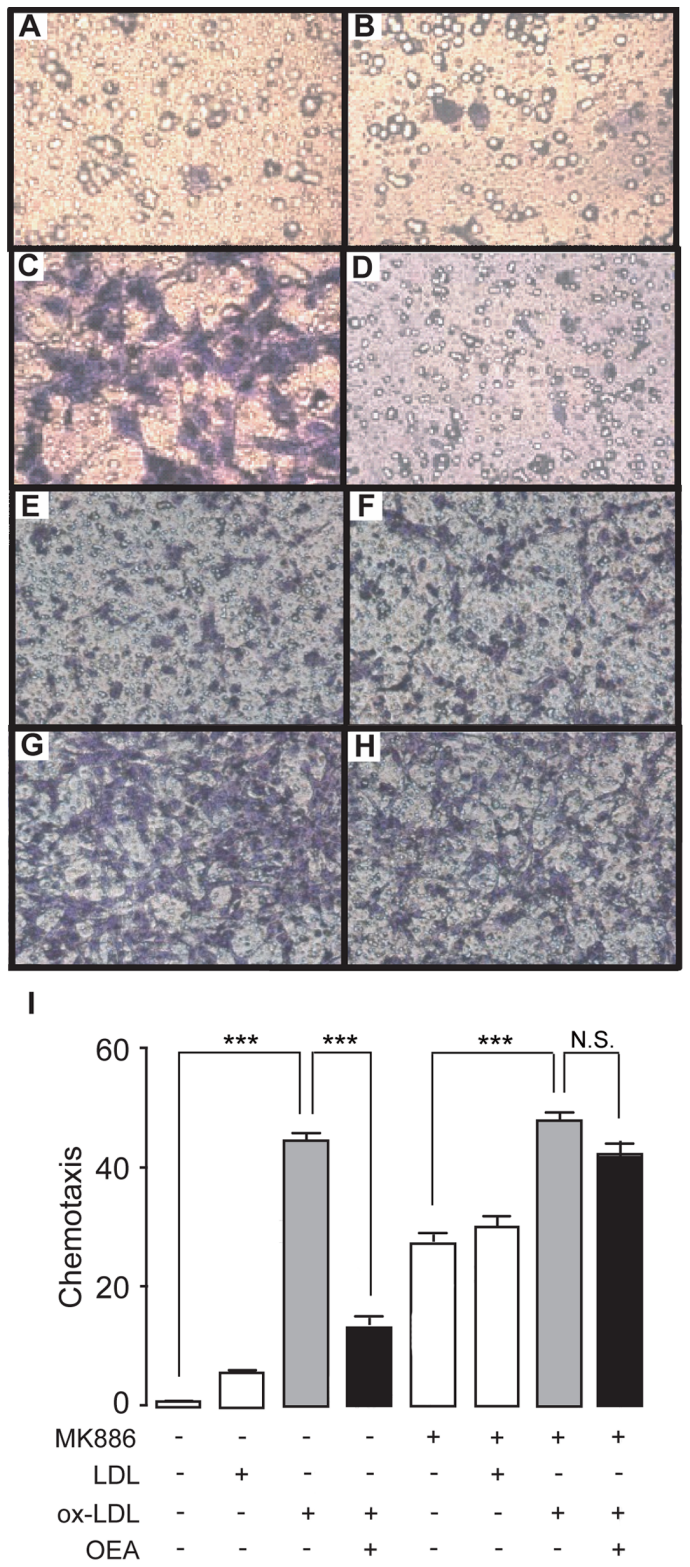
OEA reduced ox-LDL-induced VSMC migration via PPAR-α signaling. (A–H), The effect of vehicle (A), LDL (B), ox-LDL (C) ,OEA (D) and MK886 (E–H) on VSMC migration, assessed by transwell assay; (I) Quantitation of chemotaxis of A–H. Vehicle, 0.1% DMSO; LDL, 50 μg/ml; ox-LDL, 50 μg/ml; OEA, 50 μM; MK886, 10 μM. *** p<0.001, one-way ANOVA, n = 6.

Next we examined the effect of OEA on VSMC migration by trans-well assay. Rat VSMC cells were placed on the trans-well membrane inserted in 12-well plates, then treated by vehicle, LDL (50 μg/ml), ox-LDL (50 μg/ml), OEA (50 μM) with or without MK886 (10 μM) for 12 hrs. There was no significant difference on VSMC cell migration between LDL treatment and vehicle treatment (Fig. 2A–B, E–F). Compared to LDL treatment, ox-LDL caused greater VSMC cell migration (Fig. 2A–C, E–G, I), which could be blocked by OEA (Fig. 2D, I) in dose-dependent manner (Fig. S2). The inhibitory effect of OEA on VSMC cell migration was completely blocked by PPAR-α antagonist MK886 (10 μM) (Fig. 2D, H, I).

### Anti-oxidative effect of OEA was mediated by PPAR-α signal

**Figure 3:**
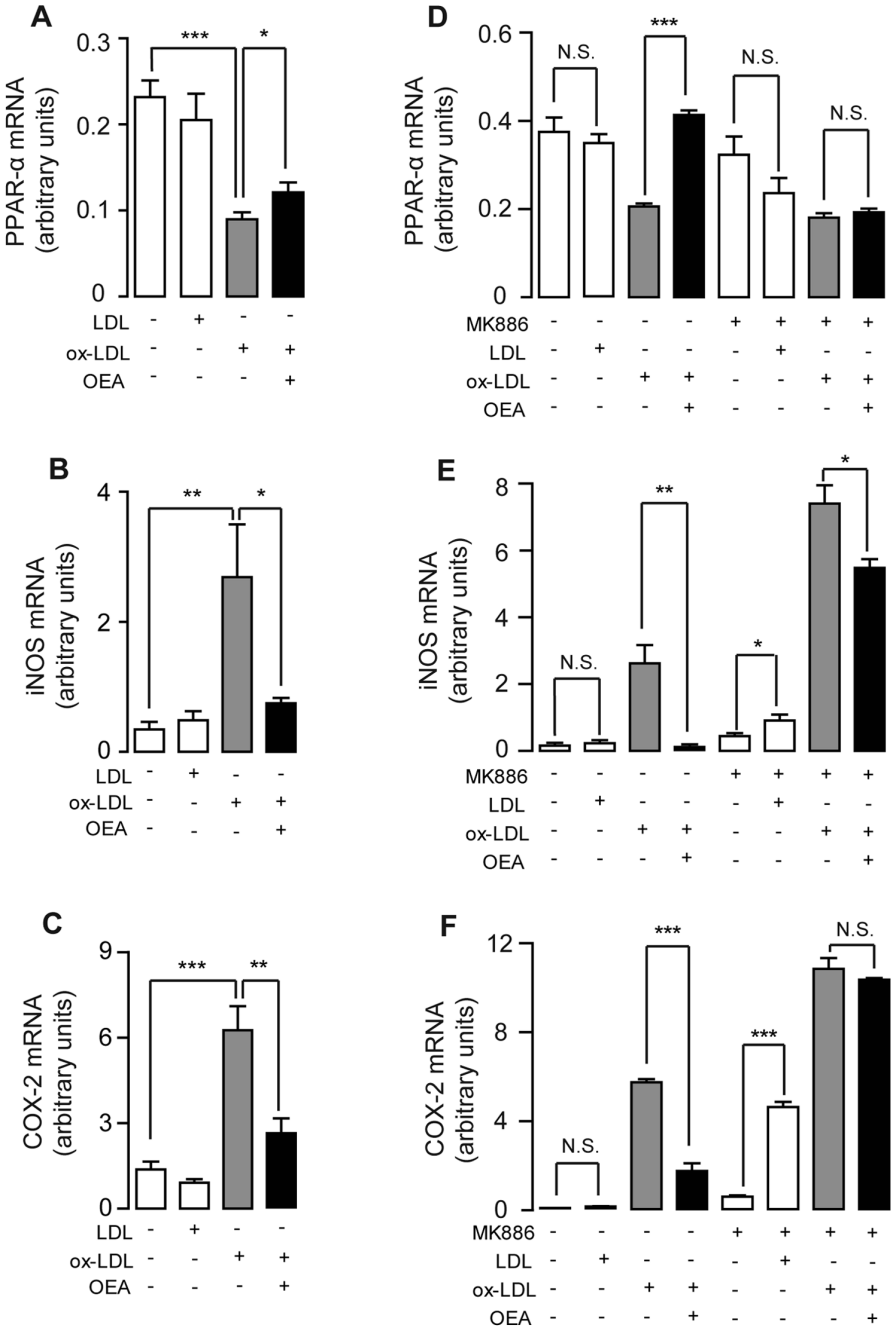
Inhibition of ox-LDL-induced inflammation by OEA was mediated by PPAR-α signaling. The effect of vehicle (0.1% DMSO), LDL (50 μg/ml), ox-LDL (50 μg/ml), OEA (50 μM) or MK886 (10 μM) on mRNA expression levels of PPAR-α (A, D), iNOS (B, E) and COX-2 (C, F) in mouse primary macrophages (A–C) or mouse macrophage RAW246.7 cells (D–F). N.S., not significant; * p<0.05, ** p<0.01, *** p<0.001, one-way ANOVA, n = 4–8.

To investigate whether OEA's anti-inflammatory properties contribute to its atheroprotective effect, we isolated mouse primary macrophages and treated them by LDL (50 μg/ml) or ox-LDL (50 μg/ml) with or without OEA (50 μM). Real-time quantitative PCR analysis demonstrated that ox-LDL treatment reduced the mRNA expression of PPAR-α (Fig. 3A), along with an increased expression of PPAR-α controlled targets, including iNOS and COX-2 (Fig. 3B, C), the two major genes involved in intracellular oxidation. However, these changes in PPAR-α and its targets induced by ox-LDL were reversed by OEA in primary macrophages (Fig. 3A–C). In contrast to ox-LDL, LDL exerted no effect on PPAR-α and its signaling pathway under the physiological condition (Fig. 3A–C). Similar results were obtained in mouse macrophage RAW264.7 cells.

On RAW264.7 macrophages, real-time quantitative PCR analysis suggested that ox-LDL treatment also reduced the mRNA expression of PPAR-α (Fig. 3D), while increased the expression of PPAR-α controlled targets, iNOS and COX-2 (Fig. 3E–F). Similarly, OEA corrected the down-regulation of PPAR-α mRNA induced by ox-LDL treatment (Fig. 3D), which was further confirmed on the protein level in the following immunoblotting study (Fig. S3). Although ox-LDL did not demonstrate down-regulation of the PPAR-α protein level (Fig. S3), however, OEA was able to markedly increase the PPAR-α protein levels in RAW264.7 microphages (Fig. S3). In addition, OEA also repressed iNOS and COX-2 mRNA expression, either at basal level (Fig. S4) or induced by ox-LDL (Fig. 3E–F) on RAW264.7 microphages. To further identify whether OEA's protective effect against oxidation is mediated by PPAR-α signaling, RAW264.7 macrophages were treated with MK886 (10 μM), a potent PPAR-α antagonist, 30 mins before ox-LDL challenges. Data showed that MK886 was able to block OEA's counteracting effects on PPAR-α, iNOS and COX-2 expression levels induced by ox-LDL on macrophages (Fig. 3D–F). Moreover, on RAW264.7 cells with LDL treatment alone, PPAR-α signaling antagonism by MK886 elevated iNOS and COX-2 mRNA expressions, while no such effect was observed in cells without MK886 treatment (Fig. 3B–C, E–F). Furthermore, compared to the cells without MK886 treatment, ox-LDL induced a greater increase of iNOS and COX-2 expression in MK886-treated cells (Fig. 3E, F). These observations suggested that PPAR-α signaling be necessary for the physiological regulation of anti-oxidation process.

### OEA reversed LPS-induced LDL modification through PPAR-α

**Figure 4:**
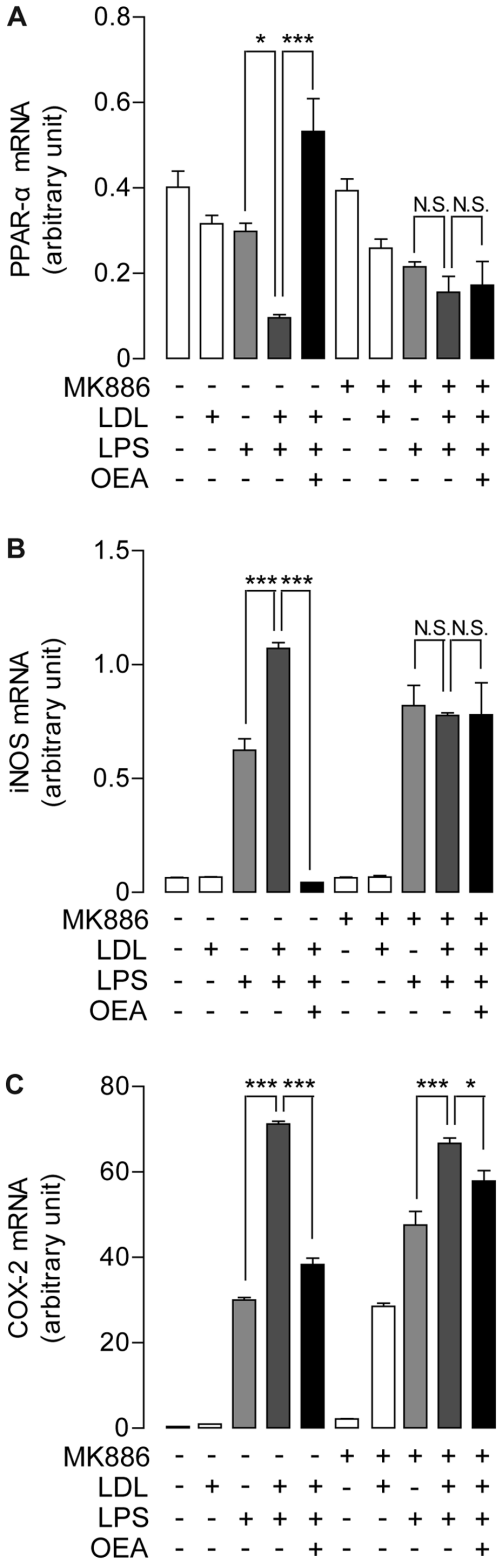
OEA suppressed LPS-induced inflammation mediated through PPAR-α signaling. The effect of OEA combined with or without MK886 on mRNA expression levels of PPAR-α (A), iNOS (B) and COX-2 (C), assessed by real-time quantitative PCR, on RAW246.7 cells treated with LPS, LDL or LPS/LDL. Vehicle, saline; LPS, 0.5 μg/ml; LDL, 50 μg/ml; OEA, 50 μM; MK886, 10 μM. * p<0.05, *** p<0.001, one-way ANOVA, n = 6.

To further investigate whether OEA modulated the inflammatory response mediated by LDL modification, we induced the inflammation in RAW264.7 cells by LPS (0.5 μg/ml) for 6 hrs, followed by LDL (50 μg/ml) treatment. As expected, LPS initiated LDL oxidation to produce ox-LDL and this process can be blocked by various concentrations of OEA (Fig. S5). Co-administration of LDL and LPS also down-regulated the PPAR-α mRNA level (Fig. 4A), an effect similar to ox-LDL treatment alone (Fig. 3A, D). However, OEA treatment restored PPAR-α expression level in dose-dependent manner (Fig. 4A, Fig. S6A). This effect of OEA was completely blocked by PPAR-α antagonist MK886 (Fig. 4A). Moreover, LPS stimulation had already induced the expressions of iNOS (Fig. 4B) and COX2 (Fig. 4C), but LDL was able to further enhance the expressions of these PPAR-α controlled genes (Fig. 4B–C), compared to the group with LPS treatment alone. Consistent with the previous work, the increased mRNA expressions of said oxidative genes stimulated by LPS and LDL were reversed by OEA (Fig. 4B–C) in dose-dependent manner (Fig. S6B–C). This effect of OEA was also acting through PPAR-α signaling, which was blocked by MK886 administration (Fig. 4B–C).

### OEA prevented the formation of atherosclerotic plaques in aorta lesion rats

**Figure 5:**
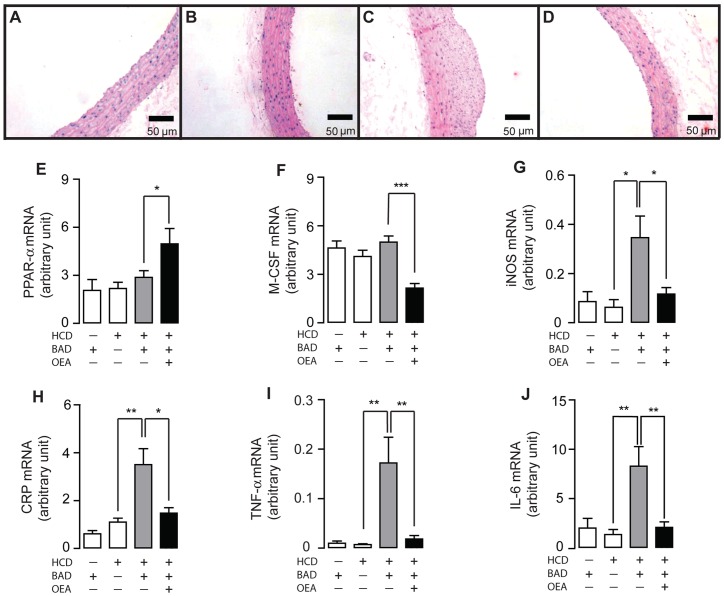
OEA prevented atherosclerotic plaque formation in BAD rats. (A–D), The effect of vehicle (A–C) or OEA (D) on atherosclerotic plaque formation in BAD rats fed with normal diet (BAD-ND, A), sham-operated rats fed with high-caloric diet (sham-HCD, B), and BAD rats fed with HCD (BAD-HACD, C-D). Scale bar, 50 μm. (E–J), The effect of vehicle (−) or OEA (+) on mRNA expression levels of PPAR-α (E), M-CSF (F), iNOS (G), CRP (H), TNF-α (I) and IL-6 (J) in aorta tissues of BAD-ND rats, sham-HCD rats and BAD-HCD rats. Vehicle, 5% PEG/5% Tween-80 in saline; OEA, 5 mg/kg/day, i.p.; * p<0.05, ** p<0.01, *** p<0.001, one-way ANOVA, n = 7–9 rats/group.

Rats were undergone balloon aortic denudation (BAD) or sham-operated procedure, and then fed with either HCD (2% cholesterol) or normal diet (ND) along with the injection of vehicle or OEA (5 mg/kg, 17 wks, i.p). Histology studies showed that arterial atherosclerotic plaques appeared in BAD rats fed with HCD for 17 weeks (HCD-BAD rats), while no plaque was observed in BAD rats fed with normal diet (ND-BAD rats) or in sham-operated rats fed with 17-week HCD (HCD-sham rats) (Fig. 5A–C). In comparison to the vehicle administration (Fig. 5C), administration of OEA was able to prevent the formation of atherosclerotic plaque in HCD-BAD rats (Fig. 5D). Biochemistry test revealed that HCD induced hyperlipidemia, increased cholesterol, triglyceride, LDL, and decreased high density lipoprotein (HDL) in both BAD rats and sham-operated rats (Fig. S7A–D). Among HCD-BAD rats, the abnormal lipid profile induced by HCD was corrected by OEA administration (Fig. S7A–D). In agreement with atherosclerotic pathology, ox-LDL and TNF-α, two key risk factors engaged in the pathological development of atherosclerosis, were increased only in HCD-BAD rats, but not in NC-BAD rats or HCD-sham rats (Fig. S7E–F). Administration of OEA was capable to normalize, at least partially, ox-LDL content and TNF-α expression in HCD-BAD rats (Fig. S7E–F). Although Q-PCR analysis demonstrated that there was no difference in mRNA expressions of PPAR-α and M-CSF in artery tissues among ND-BAD rats, HCD-sham rats and HCD-BAD rats (Fig. 5E, F), we observed an increase of PPAR-α mRNA expression (Fig. 5E) and decrease of M-CSF mRNA expression (Fig. 5F) in HCD-BAD rat with OEA administration. We further analyzed other genes participated in the oxidation and inflammation during atherosclerotic development, including COX-2, iNOS, CPR, TNF-α and IL-6, and discovered an increase of these genes in HCD-BAD rats but no changes in HCD-sham rats or NC-BAD rats (Fig. 5F–J). In HCD-BAD rats, administration of OEA completely blocked the expression of these inflammatory factors (Fig. 5F–J).

### OEA reduced the formation of atherosclerotic plaques in HCD ApoE^−/−^ mice

**Figure 6:**
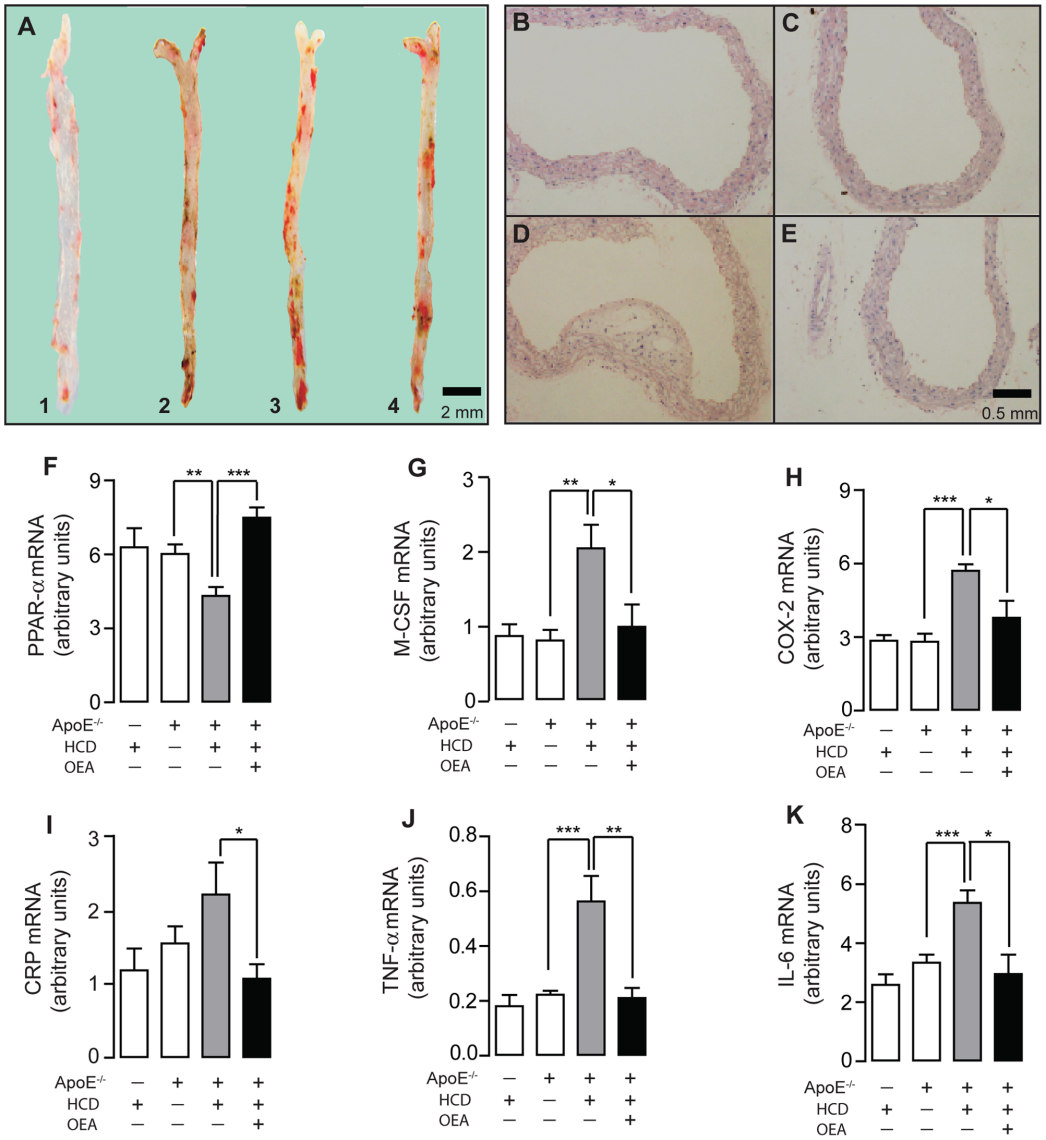
OEA reduced atherosclerotic plaque formation in ApoE^−/−^ mice. Oil red O staining (A) and H & E staining (B–E) showed aortic atherosclerotic formation in wild-type C57mice fed with HCD (A_1_, B), ApoE**^−^** ^/**−**^ mice fed with normal diet (A_2_, C), ApoE**^−^** ^/**−**^ mice fed with HCD (A_3_, D), and ApoE**^−^** ^/**−**^-HCD mice with OEA administration (5 mg/kg, i.p.) (A_4_, E). Scale bar, 2 mm or 0.5 mm. (F–K), The effect of OEA on mRNA expression levels of PPAR-α (F), M-CSF (G), COX-2 (H), CRP (I), TNF-α (J) and IL-6 (K) in aorta tissues of wt-HCD mice, ApoE**^−^** ^/**−**^-ND mice and ApoE**^−^** ^/**−**^-HCD mice. Vehicle, 5% PEG/5% Tween-80 in saline; OEA, 5 mg/kg/day, i.p.; * p<0.05, ** p<0.01, *** p<0.001, one-way ANOVA, n = 6–8 mice/group.

To further investigate OEA's anti-atherosclerotic property, we fed ApoE^−/−^ mice with HCD (HCD-ApoE^−/−^ mice) to develop atherosclerosis, and examined atherosclerotic plaque formation under OEA administration (5 mg/kg/day, 14 wks, i.p). ApoE^−/−^ mice spontaneously developed atherosclerosis after fed with HCD for 14 weeks (Fig. 6A_3_). Oil-Red-O staining (Fig. 6A) and H&E histologic staining (Fig. 6B–E) revealed an aggressive lesion development on vessel wall of HCD-ApoE^−/−^ mice (Fig. 6A_3_, D), while no obvious lesion was observed in either wild-type C57/BL6 control mice fed with HCD (HCD mice) (Fig. 6A_1_, B) or ApoE^−/−^ mice fed with normal diet (ND-ApoE^−/−^ mice) (Fig. 6A_2_, C). Comparing to vehicle administration (Fig. 6A_3_,D), OEA administration suppressed atherosclerotic plaques formation (Fig. 6A_4_, E) in HCD-ApoE^−/−^ mice. In addition, HCD-ApoE^−/−^ mice showed significantly decreased mRNA expression of PPAR-α (Fig. 6F), accompanied by the increased mRNA expressions of M-CSF, COX-2, CRP, TNF-α and IL-6 genes (Fig. 6G–K), when compared with ND-ApoE^−/−^ mice. OEA administration in HCD-ApoE^−/−^ mice was able to completely reverse the changes in the expressions of PPAR-α and its targets induced by HCD in ApoE^−/−^ mice (Fig. 6F–K). Serum biochemical analysis revealed that a significant increase of cholesterol, triglyceride, LDL and ox-LDL was observed in the HCD-ApoE^−/−^ mice, in comparison to the control mice (Fig. S8A–D). This imbalance of lipid profile can also be antagonized by OEA administration (Fig. S8A–D).

## Discussion

As oxidized LDL is considered an essential factor in atherosclerosis development, many therapeutical interventions targeting the arterial wall disorder has focused on blocking LDL oxidation. In this study, we demonstrated that LDL oxidation was a primary event in atherogenesis and the majority of LDL was modified into ox-LDL during the course of inflammation. Activation of PPAR-α by OEA, an endogenous and potent PPAR-α ligand, was able to suppress ox-LDL production in mouse macrophages and reverse the pathological effects induced by ox-LDL in atherosclerosis animals, including atherosclerotic plaque formation and inflammation. Our results indicated that OEA's anti-atherosclerosis function occurred through a transcriptional mechanism pertaining to PPAR-α signaling and its performance required the presence of a functional PPAR-α signaling pathway.

Recent evidences suggest that PPAR-α play an important role in controlling vascular cell proliferation and migration [19], [21], [31], [32]. Activation of vascular cell proliferation is a key event during the course of atherosclerotic development. In response to vascular induction, smooth muscle cells migrate into the intima of arterial wall, where they subsequently proliferate and synthesize extracellular matrix, and render intimal hyperplasia [33]. PPAR-α is present in vascular cells, including endothelial cells and smooth muscle cells, suggesting that it may have a role in regulating vascular functions. Evidences as follows indicate that PPAR-α activation is involved in vascular cell remodeling, which plays a protective role against atherosclerosis: (i) classic PPAR-α agonist fenofibrate reduced vascular endothelial cell proliferation and smooth muscle cell migration by disrupting the cell cycle [21], [31], [32]; and (ii) the induction of smooth muscle cell proliferation and migration was observed in PPAR-α knockout mice [21]. Similar to other PPAR-α agonist, OEA suppresses the abnormal endothelial proliferation and vascular smooth cell migration induced by ox-LDL challenge. Taking together, our findings confirm that PPAR-α activators can prevent the development of atherosclerosis through the inhibition of endothelial proliferation and vascular smooth muscle cell migration.

Atherosclerosis is a major complication of metabolic syndrome associated with cytokine imbalance and is now considered a chronic inflammatory disease [34]. Many studies have demonstrated that PPAR-α activators have pleiotropic effects independent on lipid modulation during atherosclerotic development, such as inflammation, plaque formation and stability, suggesting that PPAR-α may exert the anti-atherosclerotic actions directly on the vascular wall. The importance and significance of the anti-inflammatory action mediated by PPAR-α signaling in vascular cells has been revealed in recent years [35]–[37]. Though the direct effects of PPAR-α on endothelial cells and vascular smooth cells are important, its effect on macrophages is crucial during inflammation. Macrophages can be activated by a variety of pathological stimuli, e.g. ox-LDL, and produce a programed cytokine response engaged in atherosclerotic development. For instance, ox-LDL particles react with cell membrane receptors, leading to the release of cytokines, such as IL-6 and TNF-α, which promote the early development of plaque. Therefore, suppression of inflammation by reducing cytokine release may be an effective strategy in modulating the cascade of responses during atherosclerotic development [38]. Activation of PPAR-α can inhibit the expression of genes coding for various inflammatory cytokines [39], indicating PPAR-α may have a significant anti-inflammatory role. This is further supported by our data showing that activation of PPAR-α by OEA suppressed the mRNA expression of pro-inflammatory factors, such as iNOS, COX-2, IL-6, CRP and TNF-α, and that PPAR-α signaling is essential for OEA's anti-inflammation property in macrophages, which can be completely abolished by PPAR-α antagonist MK886. In concert with our finding, the clinical relevance studies revealed that PPAR-α agonist fenofibrate decreased plasma concentration of IL-6 and TNF-α in patients suffering atherosclerosis [40].

The roles of PPAR-α in regulating lipid homeostasis are well documented [41]. Activation of PPAR-α increases tissue-specific expression of target genes involved in fatty acid uptake and β-oxidation [42], [43]. PPAR-α agonists fibrates are the most potent triglyceride-lowering drugs available so far, and they can significantly increase high-density lipoprotein cholesterol [44]. However, the efficacy of fibrates in therapy preventing cardiovascular atherosclerosis remains controversial [45]. Our data demonstrated that OEA was able to effectively prevent plaque formation through anti-inflammatory action and modulate serum LDL and HDL contents in atherosclerosis animals, indicating the atheroprotective roles of PPAR-α and its agonist OEA. In addition, clinical studies have proved the beneficial effects of fibrates in therapies against cardiovascular disease [46]–[48]. For example, the Veterans Affair High-Density Lipoprotein Cholesterol Intervention Trial Study Group [48] has clearly shown that gemfibrozil reduced the risk of major cardiovascular events in patients with low level high-density lipoprotein cholesterol. In conclusion, this study suggests that OEA, a potent endogenous PPAR-α ligand, may be an effective anti-atherosclerotic agent acting through the inhibition of oxidation, inflammation and hyperlipidemia.

## Supporting Information

Figure S1 **Ox-LDL induced vascular endothelial cell proliferation or apoptosis time-dependently.** HUVEC viability was evaluated by CCK8 proliferation assay at 0, 12, 24, 36 and 48 hrs; cell apoptosis was assessed by flow cytometer at 0, 24 and 48 hrs. ox-LDL, 50 μg/ml.
(PDF)

Figure S2 **OEA dose-dependently reduced ox-LDL-induced VSMC migration.** (A–F), The effect of vehicle (A), LDL (B), ox-LDL (C), OEA (D–F) on VSMC cell migration was assessed by transwell assay; (G), Quantitation of chemotaxis of A–F. Vehicle, 0.1% DMSO; LDL, 50 μg/ml; ox-LDL, 50 μg/ml; OEA, 25 μM (D), 50 μM (E), 100 μM (F). *** p<0.001, one-way ANOVA, n = 6.
(PDF)

Figure S3 **OEA up-regulated the PPAR-α protein expression.** PPAR-α protein levels of RAW264.7 cells after vehicle, ox-LDL, ox-LDL+OEA, and ox-LDL+OEA+MK886 treatment was assessed by Western blot, followed by density analysis using Quantity One. Vehicle, 0.1% DMSO; ox-LDL, 50 μg/ml; OEA 50 μM; MK886, 10 μM.
(PDF)

Figure S4 **OEA reduced the basal expression levels of iNOS and COX-2 on RAW264.4 macrophages.** iNOS (A) and COX-2 (B) mRNA levels treated with OEA on RAW246.7 cell was assessed by real-time quantitative PCR. Vehicle, 0.1% DMSO; OEA, 10 μM, 50 μM. * p<0.05, *** p<0.001, one-way ANOVA, n = 6.
(PDF)

Figure S5 **OEA dose-dependently suppressed LPS-induced-LDL modification.** LPS-induced LDL modification was dose-dependently blocked by OEA, as assessed by ELISA. Vehicle, saline; LDL, 50 μg/ml; LPS, 0.5 μg/ml; OEA, 25 μM, 50 μM, 100 μM. *** p<0.001, one-way ANOVA, n = 6.
(PDF)

Figure S6 **OEA dose-dependently normalized gene expression levels stimulated by LPS-induced-LDL modification.** The effect of OEA on mRNA levels of PPAR-α (A), iNOS (B) and COX-2 (C) was assessed by real-time quantitative PCR on RAW246.7 cells after LDL+LPS treatment. Vehicle, saline; LDL, 50 μg/ml; LPS, 0.5 μg/ml; OEA, 25 μM, 50 μM, 100 μM. * p<0.05, ** p<0.01, *** p<0.001, one-way ANOVA, n = 6.
(PDF)

Figure S7 **OEA corrected lipid profile in atherosclerosis BAD rats.** Effect of OEA on blood plasma levels of Cholesterol (A), Triglyceride (B), LDL (C), HDL (D), ox-LDL (E), and TNF-α (F) in BAD-ND rats, sham-HCD rats and BAD-HCD rats. Vehicle, 5% PEG/5% Tween-80 in saline; OEA, 5 mg/kg/day, i.p. * p<0.05, ** p<0.01, *** p<0.001 one-way ANOVA. N = 7-9/group.
(PDF)

Figure S8 **OEA modulated lipid panel in ApoE^−/−^-HCD mice.** Effect of OEA on blood plasma lipid levels of Cholesterol (A), Triglyceride (B), LDL (C), and ox-LDL (D) in wt-HCD mice, ApoE^−/−^-ND mice, ApoE^−/−^-HCD mice. Vehicle, 5% PEG/5% Tween-80 in saline; OEA, 5 mg/kg/day, i.p; * p<0.05, ** p<0.01, *** p<0.001 one-way ANOVA. N = 6–8/group.
(PDF)

Table S1 **Primers for Q-PCR analysis.**
(DOCX)
