# Supplementary figures and images for: Atheroprotective Effect of Oleoylethanolamide (OEA) Targeting Oxidized LDL
Source: PLoS One. 2014 Jan 20;9(1):e85337. doi: 10.1371/journal.pone.0085337 (PMC3896367; doi:10.1371/journal.pone.0085337)

**Figure S1**

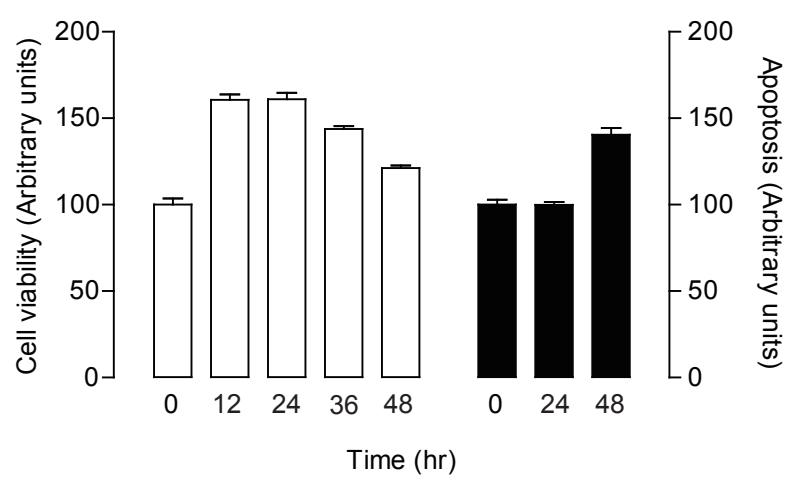

Supplement: Figure S1 — Ox-LDL induced vascular endothelial cell proliferation or apoptosis time-dependently. HUVEC viability was evaluated by CCK8 proliferation assay at 0, 12, 24, 36 and 48 hrs; cell apoptosis was assessed by flow cytometer at 0, 24 and 48 hrs. ox-LDL, 50 μg/ml. (PDF) [file pone.0085337.s001.pdf]

Figure S2

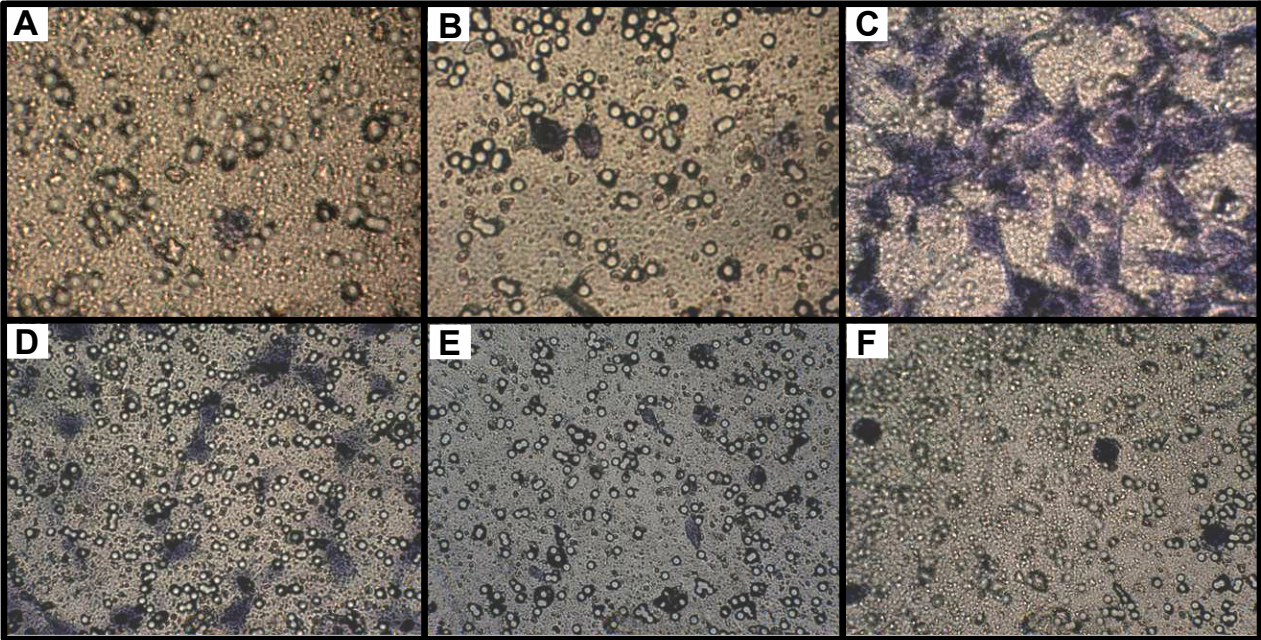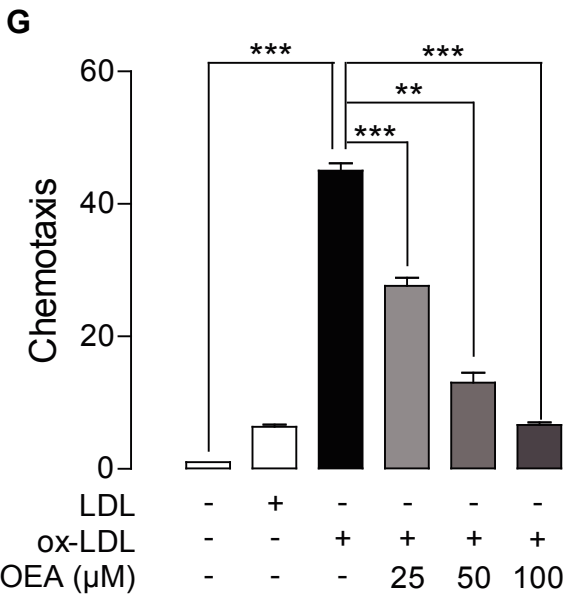

Supplement: Figure S2 — OEA dose-dependently reduced ox-LDL-induced VSMC migration. (A–F), The effect of vehicle (A), LDL (B), ox-LDL (C), OEA (D–F) on VSMC cell migration was assessed by transwell assay; (G), Quantitation of chemotaxis of A–F. Vehicle, 0.1% DMSO; LDL, 50 μg/ml; ox-LDL, 50 μg/ml; OEA, 25 μM (D), 50 μM (E), 100 μM (F). *** p<0.001, one-way ANOVA, n = 6. (PDF) [file pone.0085337.s002.pdf]

**Figure S3**

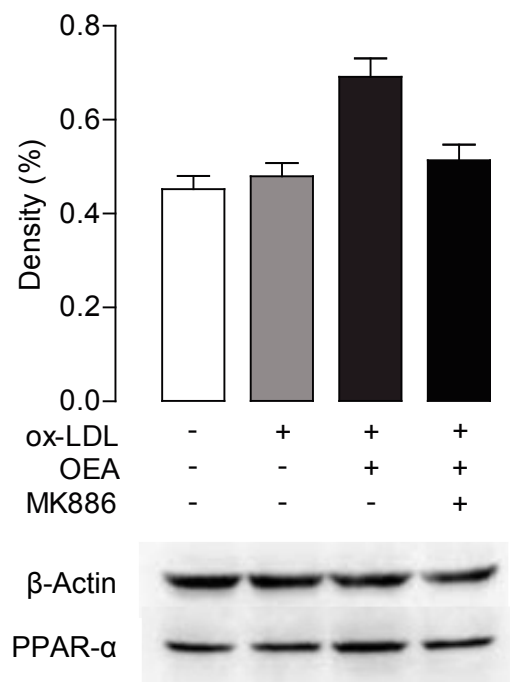

Supplement: Figure S3 — OEA up-regulated the PPAR-α protein expression. PPAR-α protein levels of RAW264.7 cells after vehicle, ox-LDL, ox-LDL+OEA, and ox-LDL+OEA+MK886 treatment was assessed by Western blot, followed by density analysis using Quantity One. Vehicle, 0.1% DMSO; ox-LDL, 50 μg/ml; OEA 50 μM; MK886, 10 μM. (PDF) [file pone.0085337.s003.pdf]

Figure S4

A

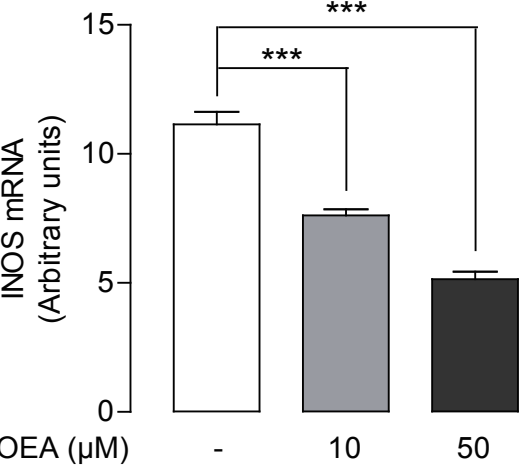

B

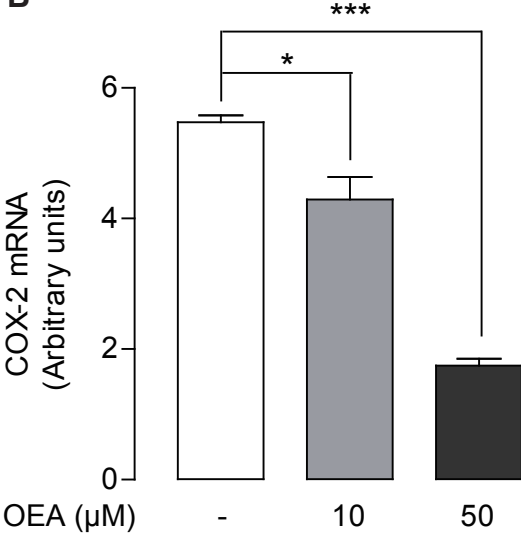

Supplement: Figure S4 — OEA reduced the basal expression levels of iNOS and COX-2 on RAW264.4 macrophages. iNOS (A) and COX-2 (B) mRNA levels treated with OEA on RAW246.7 cell was assessed by real-time quantitative PCR. Vehicle, 0.1% DMSO; OEA, 10 μM, 50 μM. * p<0.05, *** p<0.001, one-way ANOVA, n = 6. (PDF) [file pone.0085337.s004.pdf]

**Figure S5**

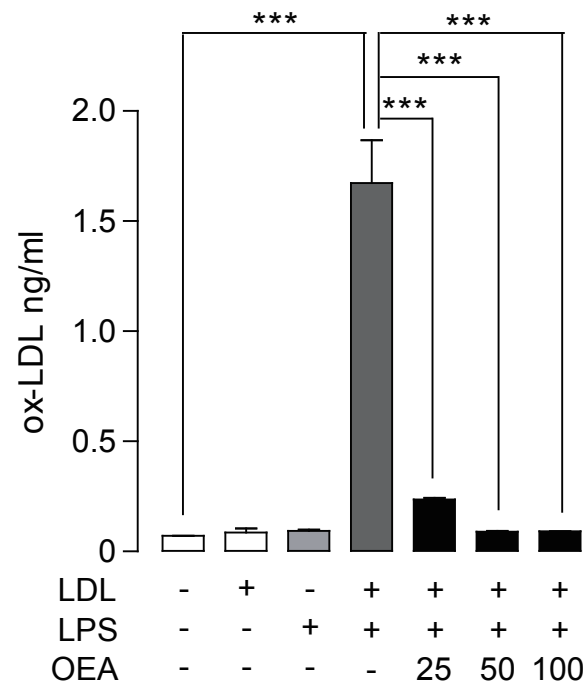

Supplement: Figure S5 — OEA dose-dependently suppressed LPS-induced-LDL modification. LPS-induced LDL modification was dose-dependently blocked by OEA, as assessed by ELISA. Vehicle, saline; LDL, 50 μg/ml; LPS, 0.5 μg/ml; OEA, 25 μM, 50 μM, 100 μM. *** p<0.001, one-way ANOVA, n = 6. (PDF) [file pone.0085337.s005.pdf]

Figure S6

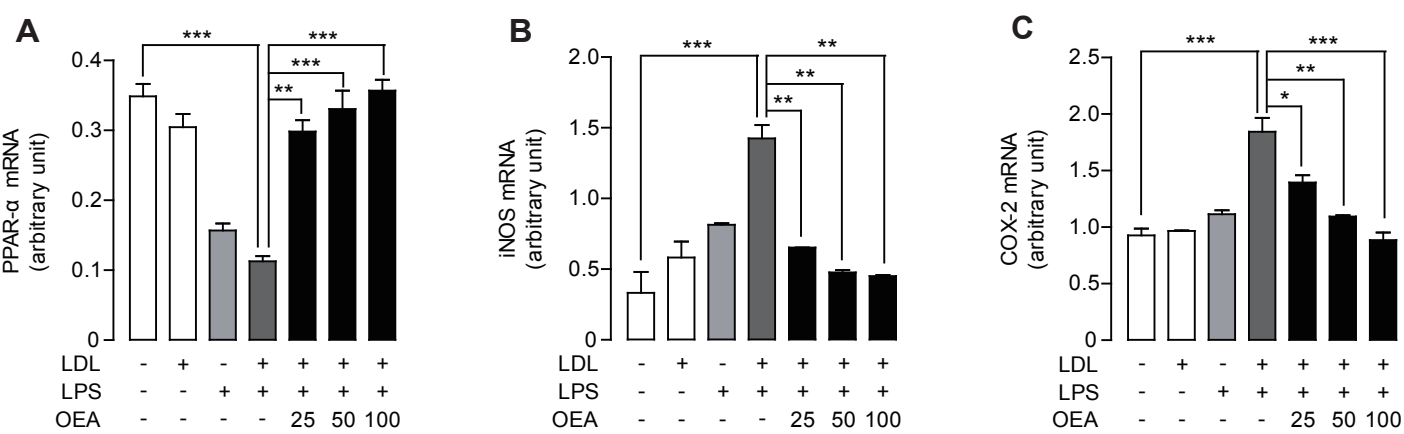

Supplement: Figure S6 — OEA dose-dependently normalized gene expression levels stimulated by LPS-induced-LDL modification. The effect of OEA on mRNA levels of PPAR-α (A), iNOS (B) and COX-2 (C) was assessed by real-time quantitative PCR on RAW246.7 cells after LDL+LPS treatment. Vehicle, saline; LDL, 50 μg/ml; LPS, 0.5 μg/ml; OEA, 25 μM, 50 μM, 100 μM. * p<0.05, ** p<0.01, *** p<0.001, one-way ANOVA, n = 6. (PDF) [file pone.0085337.s006.pdf]

**Figure S7**

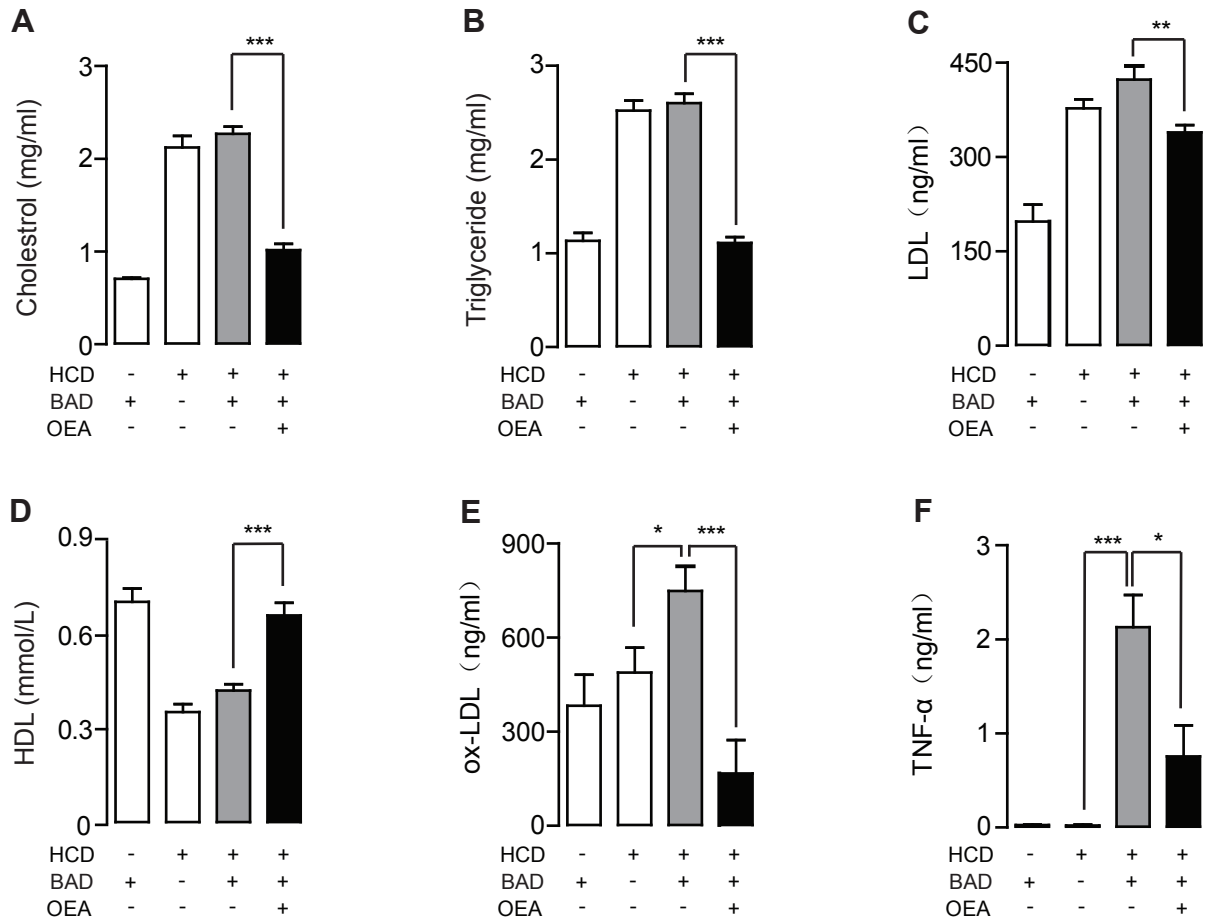

Supplement: Figure S7 — OEA corrected lipid profile in atherosclerosis BAD rats. Effect of OEA on blood plasma levels of Cholesterol (A), Triglyceride (B), LDL (C), HDL (D), ox-LDL (E), and TNF-α (F) in BAD-ND rats, sham-HCD rats and BAD-HCD rats. Vehicle, 5% PEG/5% Tween-80 in saline; OEA, 5 mg/kg/day, i.p. * p<0.05, ** p<0.01, *** p<0.001 one-way ANOVA. N = 7-9/group. (PDF) [file pone.0085337.s007.pdf]

**Figure S8**

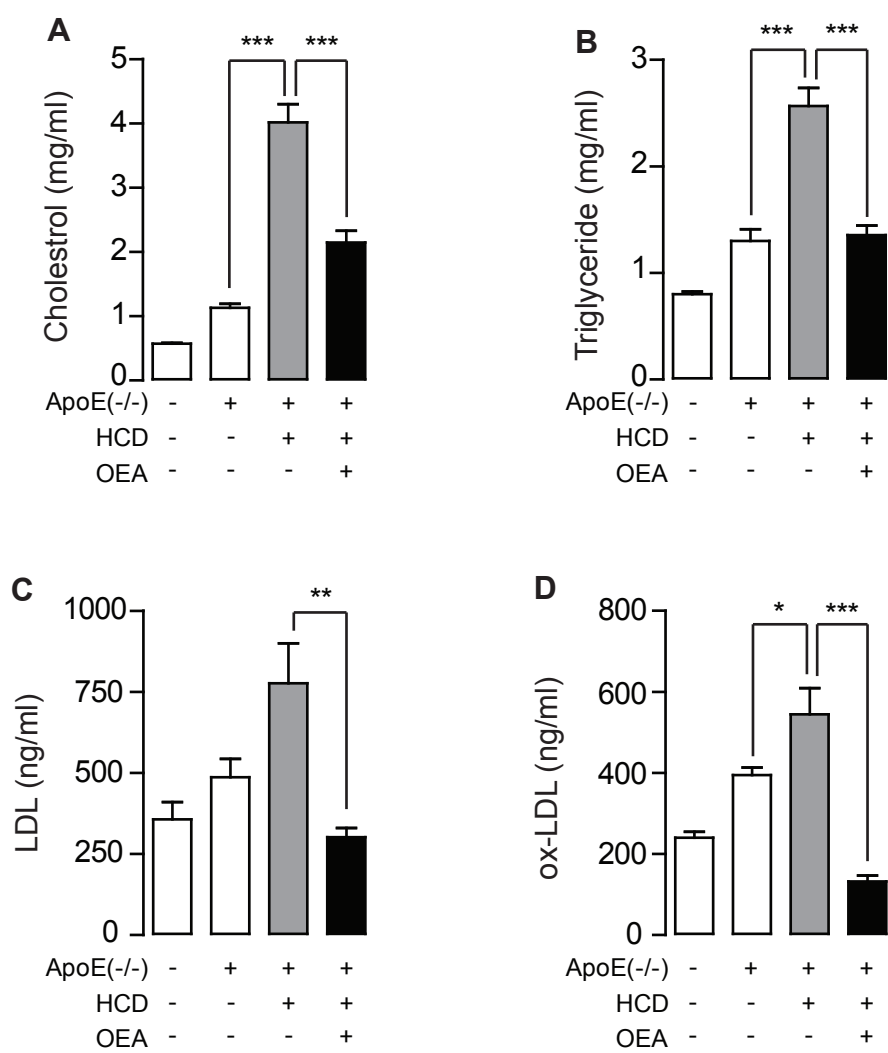

Supplement: Figure S8 — OEA modulated lipid panel in ApoE−/−-HCD mice. Effect of OEA on blood plasma lipid levels of Cholesterol (A), Triglyceride (B), LDL (C), and ox-LDL (D) in wt-HCD mice, ApoE−/−-ND mice, ApoE−/−-HCD mice. Vehicle, 5% PEG/5% Tween-80 in saline; OEA, 5 mg/kg/day, i.p; * p<0.05, ** p<0.01, *** p<0.001 one-way ANOVA. N = 6–8/group. (PDF) [file pone.0085337.s008.pdf]
