# Supplementary material for: Atheroprotective Effect of Oleoylethanolamide (OEA) Targeting Oxidized LDL
Source: PLoS One. 2014 Jan 20;9(1):e85337. doi: 10.1371/journal.pone.0085337 (PMC3896367; doi:10.1371/journal.pone.0085337)
Supplement: Table S1 — Primers for Q-PCR analysis. (DOCX) [file pone.0085337.s009.docx]

| Table S1. Primers for Q-PCR analysis | | |
| --- | --- | --- |
| **Gene** | **Forward Sequence** | **Reverse Sequence** |
| mPPAR-α | ACGATGCTGTCCTCCTTGATG | GTGTGATAAAGCCATTGCCGT |
| mM-CSF | AAAGAAGCCCTGAACCTCCTGGAT | AGCAGCAGTCTGAGAAGCTGGATT |
| mCRP | GGGTGGTGCTGAAGTACGAT | AAACATTGGGGCTGAGTGTC |
| mTNF-α | AATGGCCTCCCTCTCATCAGTTCT | TGAGATAGCAAATCGGCTGACGGT |
| mIL-6 | AATTAAGCCTCCGACTTGTGAAG | CTTCCATCCAGTTGCCTTCTTG |
| miNOS | CCCGTCCACAGTATGTGAGGAT | CATTACCTAGAGCCGCCAGTGA |
| mCOX-2 | CCAGCAAAGCCTAGAGCAAC | AGCACAAAACCAGGATCAGG |
| mGAPDH | TGTGTCCGTCGTGGATCTGA | TTGCTGTTGAAGTCGCAGGAG |
| rPPAR-α | TGGTGGACCTCCGGCA | TCTTCTTGATGACCTGCACGA |
| rM-CSF | ACAGAGCAACCAAATCACGAGG | CCCAGTTAGTGCCCATTGAAGA |
| rCRP | TGTCTCTATGCCCACGCTGATG | GGCCCACCTACTGCAATACTAAAC |
| rTNF-α | GGCTCCCTCTCATCAGTTCCA | CGCTTGGTGGTTTGCTACGA |
| rIL-6 | TGCCTTCTTGGGACTGATGTTG | TGGTCTGTTGTGGGTGGTATCC |
| riNOS | TGGTGGTGACAAGCACATTT | CTGAGTTCGTCCCCTTCTCC |
| rCOX-2 | GCACAAATATGATGTTCGCATTCT | GAACCCAGGTCCTCGCTTCT |
| rGAPDH | AAGTATGATGACATCAAGAAGGTGGT | AGCCCAGGATGCCCTTTAGT |
